# The Research on Web-Based Testing Environment Using Simulated Annealing Algorithm

**DOI:** 10.1155/2014/167124

**Published:** 2014-05-14

**Authors:** Peng Lu, Xiao Cong, Dongdai Zhou

**Affiliations:** ^1^Department of Media Technology and Communication, Northeast Dianli University, Jilin, Jilin 132012, China; ^2^College of Science, Northeast Dianli University, Jilin, Jilin 132012, China; ^3^School of Software, Northeast Normal University, Jilin, Changchun 130117, China

## Abstract

The computerized evaluation is now one of the most important methods to diagnose learning; with the application of artificial intelligence techniques in the field of evaluation, the computerized adaptive testing gradually becomes one of the most important evaluation methods. In this test, the computer dynamic updates the learner's ability level and selects tailored items from the item pool. In order to meet the needs of the test it requires that the system has a relatively high efficiency of the implementation. To solve this problem, we proposed a novel method of web-based testing environment based on simulated annealing algorithm. In the development of the system, through a series of experiments, we compared the simulated annealing method and other methods of the efficiency and efficacy. The experimental results show that this method ensures choosing nearly optimal items from the item bank for learners, meeting a variety of assessment needs, being reliable, and having valid judgment in the ability of learners. In addition, using simulated annealing algorithm to solve the computing complexity of the system greatly improves the efficiency of select items from system and near-optimal solutions.

## 1. Introduction

In recent years, with the rapid development of computer and Internet technology, the interaction between teachers and students is constantly enriched; it has developed more and more e-learning environment for instructor courses and evaluation of learners [1–4] and it provides a convenient, flexible, and interactive learning environment between teachers and students, making the learning of learners more flexible that you can learn anytime and anywhere [5–8]. In order to more accurately grasp the learner's learning and easily provide the best learning content for them [9], more and more studies are used in artificial intelligence [10–12], data mining techniques [13–16], and fuzzy theory [17, 18] to develop e-learning systems. These studies have a common goal, which is based on the learner's learning, to help them get the best learning status.

Assessment is part of the learning process; it is related to the achievement of learning outcomes [19]. In the assessment of learning outcomes, effective evaluation is an important part of the e-learning. Thus, the learning process can be identified further [20, 21]. Traditionally, teachers will use paper and pencil test to evaluate the learner's learning. With the deepening of the application of computer technology in education and teaching, more and more large-scale tests using a computer as a tool, such as GRE, IELTS, and TOEFL, are used. These forms of test are called computer-based test (CBT) and CBT with an additional way to supplement the traditional paper and pencil test [22, 23]. With respect to the paper and pencil test, the potential advantages of CBT include the following: the tests can use a variety of media that provide a more meaningful examination of the candidates, such as multimedia graphics, audio, or video; reuse of test items becomes easy; timely access to the evaluation results saves a lot of time for teachers; CBT will automatically record and store the testing process of learning in exam to facilitate further analysis. In this way, teachers can provide more useful suggestions in order to improve learning for learners. So far, the developed CBT systems mainly concentrated on the organization and construction of the item pool [24–26], automatic generation of papers [23], and so on.

Most of the teachers and students evaluation services only provide a medium but often do not provide a meaningful function for them. Therefore, teachers and students cannot get meaningful information from these platforms. The exam is one of the main goals to discover and diagnose the problems of learners in the learning process. In CBT, the linearity test is a general evaluation model, which provides the same number and same sequence of items for all students. However, this method only displays the test digitally; it looks like imitation paper and pencil test. In addition, each learner's learning style or learning methods have significant differences; therefore, the evaluation process should be used in different items according to their characteristics so that we can achieve a reasonable evaluation of them. Thus, scholars have proposed adaptive assessments, and adaptive assessments provide learners with a personalized assessment environment. Compared with the traditional test system, adaptive assessment is a dynamic assessment process [27–29]. Adaptive assessment system is often called computerized adaptive testing (CAT) [30–33]. In this form of test, the system dynamic select appropriate items that fits every learner according to the response of previous items. Specifically, on the basis of the initial situation of the learner, through the following steps the test is implemented: first, the system according to the current test results of learner and real-time search of all exam items determines which the next most appropriate item of learner is; that is, the next item is selected in dynamic based on the evaluation of learners in adaptive testing; then, the system shows the item and learner's response and then the system reevaluates its level of knowledge according to the learner's answer, repeating the above steps until the termination criteria; finally, the output of the evaluation results is provided, thus achieving a reasonable assessment of learners in science [34]. In recent years, researchers have mainly used item response theory (IRT) as a theoretical basis to develop a CAT system [35, 36]. Compared with traditional testing, each learner had a unique test, unlike conventional test systems to provide each learner the same items; the items selection in CAT is based on his/her own ability which is estimated in the test process to adapt to each learner [37–40]. The research shows that adaptive assessments are more effective than nonadaptive methods [27, 41]. And adaptive assessments provide learners with a more effective estimate of the level of ability [42, 43]. In addition, adaptive assessment does not make learners tired, because it creates a test which is suitable for learners' qualification. Therefore, the items which learner meets are both not very difficult and not very easy, and it will take the most appropriate items for the level of ability of learner [43, 44].

The item pool is not only an important part of the test but also the basis for the implementation. In order to meet the needs of the test, it must have a large number of high quality items in the item pool. In the implementation of the testing process, on the one hand, system requires real-time to search the entire item pool and calculate the amount of information based on the parameters of items and the parameters of learner's temporary ability level to achieve the purpose of selecting the appropriate items to learners; on the other hand, the system should, according to the learner's response situation of the items, reestimate the ability level of students. Therefore, with the increase in the number of items, the efficiency of the test becomes further deteriorated. Using the heuristic algorithm in an acceptable cost, such as time and space, the problem can be solved given an approximate optimal solution. Therefore, it is a feasible way to solve this problem. This can reduce the time to choose the items and improve efficiency to meet the needs of actual test.

In this study, we proposed a method which is based on simulated annealing algorithm for web-based testing environment. With this new approach, the system based on multiple criteria, including the amount of information of the items, exposure frequency, and exam topics and adaptability selects more suitable items for each learner to achieve “individualized instruction.” Importantly, by combining the simulated annealing algorithm in item selection mechanism and by selecting a suitable cooling schedule, this method can provide optimal solutions and can reduce the computational complexity and has an acceptable execution time, thereby increasing the search speed of items in large item pools and improving efficiency. After the response of the item, the system using maximum likelihood estimation method (MLE) as the underlying psychological theory estimates the ability of students and timely feedback to the student results; the final test results not only demonstrated the ability level of the students but also reflected the student's rank. In addition, teachers can also be preestablished criteria for the test to evaluate student achievement or learning performance. Therefore, teachers and students can evaluate the teaching objectives and learning outcomes through the test results. Further, the number of experiments evaluates system availability, accuracy, and efficiency. The results of the evaluation show that the web-based testing environment using simulated annealing algorithm supports individualized evaluation function. The system can be reliable and effective evaluation of the ability level of the students.

The rest of the paper is organized as follows. In Section 2, the simulated annealing algorithm was introduced. In Section 3, the problem of item selection in adaptive testing is described. The architecture of web-based testing environment in Section 4 was introduced.  Section 5 describes the experimental results. Finally, a summary and outlook are available in Section 6.

## 2. Simulated Annealing Algorithm

Kirkpatrick inspired solid annealing process, introduced the metropolis criterion into the combinatorial optimization problems, and proposed simulated anneal algorithm (SA). SA algorithm is an effective algorithm for solving large-scale combinatorial optimization problems [45]; it has high efficiency, robustness, versatility, and flexibility characteristics [46, 47]. And the cooling schedule, neighborhood structure, the new solution generator, acceptance criteria, and the random number generator together constitute the three pillars of the algorithm.

*(1) Cooling Schedule.* It includes a set of parameters to control the process of algorithm and uses it to progress the convergence state of SA when the algorithm returns an approximate optimal solution with limited execution process. Therefore, the reasonable selection of the cooling schedule is key to successful implementation algorithm. A cooling schedule includes the following parameters:

- the initial value *t*_0_ of the control parameter *t*: the values of *t*_0_ should be as large as possible in order to quickly reach a quasi-equilibrium state;
- attenuation function *t*_*k*_ of control parameter *t*: to avoid the algorithm to generate long Markov chain, the select principles of attenuation function *t*_*k*_ are “small is appropriate”;
- Markov chain length *L*_*k*_: under the premise that attenuation function has been selected, the selection of *L*_*k*_ should ensure that on each control parameter value the quasi-equilibrium can be restored;
- the final value *t*_*f*_ of the control parameter *t*: the selection of *t*_*f*_ should take into account the quality and execution time of the final solution.

*(2) Neighborhood Structure and New Solution Generator.* For each solution as *i* ∈ *S*, the existence set of solutions *S*_*i*_ ⊂ *S*. In a sense, these solutions are the “neighboring” solution of *i*; the set *S*_*i*_ is called neighborhood of *i*. And each *j* ∈ *S*_*i*_ is called a near solution of *i*. The new solution generator refers to a method of select solution *j* from the neighborhood *S*_*i*_ of the solution *i*.

*(3) Acceptance Criteria and Random Number Generator.* Use metropolis algorithm to generate a sequence of combinatorial optimization problem solution; then, according to the transition probability *P*_*t*_, decide whether to accept the transfer from the current solution *i* to new solution *j*. The transition probability *P*_*t*_ is shown in the following equation:

(1) $$\begin{array}{c} P_{t}\left(i⟹j\right)=\{\begin{array}{cc} 1, & \text{if}\;f\left(j\right)\leq f\left(i\right)\\ e^{\left((f(i)-f(j))/t\right)}, & \text{else}. \end{array} \end{array}$$

The SA algorithm from the start is in an initial solution which usually is randomly selected; then, it reduces the value of the control parameters and iterative execution metropolis algorithm, continues implementation of the iterative process of “generate new solution; judgment; accept/discard the new solution,” after converting a large number of solutions, and finds overall optimal solution for combinatorial optimization problems after ultimately the temperature *t* tends to zero. Since the beginning, the control parameter value *t* is high; it can accept a lower deterioration solution. With the value of *t* gradually decreased, the deterioration can only accept a better solution. Finally, when *t* value approaches zero, SA algorithm no longer accepts any deterioration solution. This makes the SA algorithm acceptable to deteriorate solution in a certain range and have the opportunity to escape from local optima “trap” to calculate the overall optimal solution [48].

In a typical SA algorithm, the algorithm terminates at a predetermined stop criterion; for example, in several successive Markov chains solution has not been any improvement or the error *e* of the current solution is less than the error of the provisions and so on. However, since the algorithm is a randomized search process, it can be accepted as part of the deterioration of the solution when the temperature *t* is larger, and the probability of accepting the deterioration solution decreases with the attenuation of *t*. Also, before reaching the optimal solution, in many cases it must be temporarily deteriorated solution, which is called “ridge.” The above termination criteria are not guarantees of the final solution which is just the optimal solution in the whole search process. Therefore, in order to ensure the quality of the algorithm solution, the SA algorithm adds a “memory”; with this “memory” it remembers the best result during the search, so that at the end of the annealing process the solution in the “memory” is a final result.

## 3. Description of the Problem

Next, we have the detailed description of the problem of the constraints which adaptive testing faced, the definition of item generation problem, and a memory-based simulated annealing algorithm as below.

### 3.1. Constraints

In order to meet the needs of actual testing, the adaptive testing considers the following three aspects of constraints.

*(1) Item Information.* Confidence interval of estimated trait or ability level indicates the effectiveness of the test; the IRT use of item information functions as a reference to create and test analysis and diagnosis. For different ability levels of learners, each item as reflected in the amount of information is different. The relationship between the amount of information of test and the accuracy of ability to estimate is $SE\left(\theta \right)=1/\sqrt{\sum I_{i}\left(\theta \right)}$. This relationship illustrates that the greater the amount of information of the test, the lower the length of the test and the higher the accuracy of the test.

*(2) Content Balance.* In order to conduct a comprehensive and reasonable evaluation of learner, the test must cover all the contents within the domain in practical tests. Therefore, the content of the balance control mechanism should be incorporated into the item selection process. Assuming a test target is *M* content domain (topic) and each topic has a different weight *w*_*k*_, then each item in the item pool is related to one or more topics, namely, *r*_*k*_, 1 ≤ *k* ≤ *M*. Use these relationships to achieve comprehensive evaluation of the level of ability of learners.

*(3) Item Exposure.* The traditional item generation algorithms entirely rely on the parameters information of item, and the system adaptively selects “optimal” item for learner based on the level of knowledge, so the items are likely to show the same learners as many times in the test or show the same items for most learners in the same test. This will lead to uneven distribution of item exposure and the exposure of part of the items is too high, increasing the risk of leakage questions that affect test security [49]. The main means to solve this problem is to control exposure to the items.

Through the above description of the problem, select test items not only to meet the accuracy requirements but also to meet the test comprehensive and test security needs. The item generation problem is one of the key questions in the computer adaptive testing. In this paper, we propose an item generated model which simulated annealing algorithm with memory-based algorithm.

**Definition 1**

Item Selection Problem (IGP). Let the items set *S* = {*x*_*i*_}. Item information is $I_{i}(\hat{\theta })$; the exposures of each item *x*_*i*_ in the item pool are *e*_1_, *e*_2_,…, *e*_*N*_, respectively; the test involves the number of content domain which is *M*, and the weight for each content domain is *w*_*k*_; the number of items in the test selected from each content domain is denoted by *m*_1_, *m*_2_,…, *m*_*k*_. The problem is to find an optimal item that satisfies all the constraints and makes the maximum objective function value.

### 3.2. Description of Problem Based on Simulated Annealing Algorithm

To solve this problem, the use of a memory of the simulated annealing algorithm is described as follows.

*(1) Solution Space.* We define the solution space as the set of all feasible solutions, namely, *S* = {*x*_1_, *x*_2_,…, *x*_*n*_}, where *x*_*i*_ is the *i*th item. The initial solution is randomly selected item from item pool.

*(2) The Objective Function.* The objective function is to ensure the maximum of item information, exposure control, and content balance and is defined as follows:

(2) $$\begin{array}{c} \left(\max F\left(x\right)=\alpha \cdot I+\beta \cdot B+\gamma \cdot E\right),\;x\in S, \end{array}$$

where *F*(*x*) is the objective function; parameter *I* indicates the index of information of item; parameter *B* indicates the index of content balance; parameter *E* indicates the index of exposure; parameter *α*, parameter *β*, and parameter *γ* are the weight of each index; *S* is the solution space. Descriptions of the three indicators are as follows.

*(a) Item Information.* When using the three parameters logistic model, the information function of item *i* is as follows:

(3) $$\begin{array}{c} I_{i}\left(\hat{\theta }\right)=\frac{{\left[{P_{i}}^{\prime }\left(\hat{\theta }\right)\right]}^{2}}{P_{i}\left(\hat{\theta }\right)Q_{i}\left(\hat{\theta }\right)}, \end{array}$$

where $\hat{\theta }$ is the value which is temporary estimate of the learners' knowledge level during the test and $P_{i}(\hat{\theta })$ is the probability of correct responses to item of learner.

*(b) Content Balance. *Consider

(4) $$\begin{array}{c} B=\frac{1}{e^{w_{k}m_{k}^{2}\upsilon^{-1}}},\;1\leq m\leq M. \end{array}$$

It shows the relevance of selected items and topics; parameter *υ* is a constant; parameter *e* is used to standardize.

*(c) Item Exposure.* Consider

(5) $$\begin{array}{c} E=\frac{1}{e^{n^{2}\sigma^{-1}}},\;1\leq n\leq N. \end{array}$$

The system records the number of test items in the past as *n*; parameter *σ* is a constant and parameters *e* is used to standardize.

*(3) Generate New Solution. *Based on the current* Id* of item and that as a center, *r* is the radius (as a polynomial function of the size of the items) of range *A* as the neighborhood. Then, using a random manner select from the neighborhood to the next item a new solution.

*(4) The Difference of Objective Function. *The difference of objective function with the above new solution is

(6) Δf=10·(Pi′[(θ^)]2[Pi(θ^)Qi(θ^)]−[Pj′(θ^)]2[Pj(θ^)Qj(θ^)]) +2·(1ewkmk2υ−1−1ewlml2υ−1)+3·(1eni2σ−1−1enj2σ−1).

*(5) Acceptance Criteria. *Consider

(7) $$\begin{array}{c} P=\{\begin{array}{cc} 1, & \text{if}\;\;\;\Delta f>0\\ \text{ex}{\text{p}}^{\left(\Delta f/t\right)}, & \text{else}. \end{array} \end{array}$$

*(6) Stop Criterion.* The stop criteria are several solutions that do not get any better in the successive Markov chain and the final value *t*_*f*_ of the control parameter *t* is 0.

*(7) Memory. *Use variable* BestId* as “memory” and memory of the best solution for the entire annealing process. Initially, it stores the *id* of item which is first selected by algorithm.

### 3.3. Algorithm Flow

**Figure 1:**
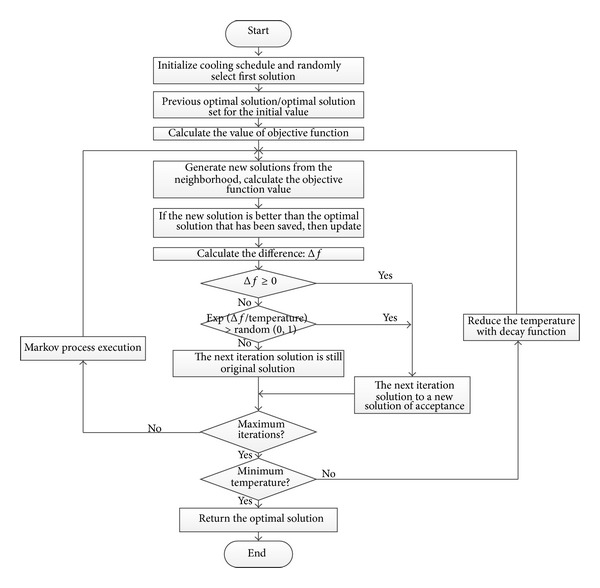
Item generation flow chart.

In the study, the objective function uses the simulated annealing algorithm with memory and order to find the approximate optimal solution in polynomial time and improve the efficiency of item selection. The flow chart of item is generated as shown in Figure 1 and includes the following steps.

**Step 1 (initialization cooling schedule)**

Let the initial temperature *T* = 100, attenuation parameters are 0.95, the length of Markov chain is 25, and termination conditions are the difference between newly generated optimal solution and the previous best solution between less threshold value *e*(0.0001).

**Step 2 (the selection of initial solution)**

Randomly select an item, take the* Id* of this item as the* Id* of optimal item;* Id* =* BestId*. And calculate the objective function value based on the* Id* of the item.

**Step 3 (the selection of next solution)**

According to* Id* of the item and randomly selected* NextId* from the neighborhood.

**Step 4 (temporary optimal solution)**

Calculate the difference between the objective function based on the new* Id*; if the objective function value of new item is greater than or equal objective function value of temporary optimal item, update and save the optimal solution's* Id* for* BestId*.

**Step 5 (acceptance criteria)**

If the difference Δ*f* which is between the objective function value of new item* NextId *and objective function value of previous item* PreId *is greater than 0, then* NextId* is accepted as a new* PredId* and the next iteration point is to accept the new point; else, take |Δ*f*| compared with a random number which is generated by a random number generator. If |Δ*f*| is larger than the random number,* NextId* is still accepted as the next iteration point. On the contrary, still take* PreId *as the next point of iteration.

**Step 6 (the Markov process)**

According to Markov chain repeat Step 3 to Step 5, when the maximum length of the Markov chains is the end of a metropolis algorithm.

**Step 7 (“slowly” annealing and find the approximate optimal solution)**

Lower the temperature and repeat Step 3 to Step 6, until the termination standard is met or attenuation function is minimized. At this point the* BestId* is searched for the approximate optimal solution.

**Step 8 (selected item)**

Finally, according to* BestId* selected items and it is presented to the candidates for test.

### 3.4. Code Description

Based on the above ideas of the simulated annealing algorithm, the code describes item generation problem as shown in Algorithm 1.

## 4. Web-Based Testing Environment

The web-based testing environment proposed in this study can be intelligent select items which are appropriate to the level of knowledge of learner through the system, as well as reasonable evaluation of learners. Next, the architecture of the environment and adaptive testing procedure are described in detail.

### 4.1. Architecture

**Figure 2:**
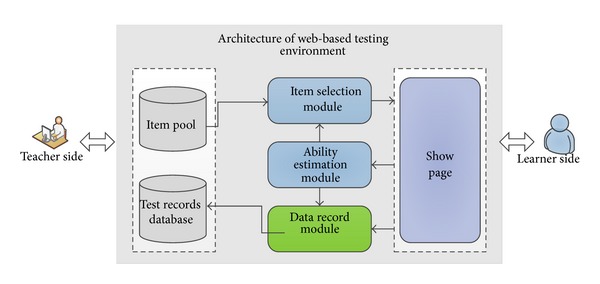
The architecture of web-based testing environment.

Figure 2 shows the architecture of web-based testing environment, which consists of the following components.

*(1) Response Model.* Use the three-parameter logistic item response models building item pools and estimated knowledge level of the learners. The results obtained do not depend on the tools used.

*(2) Item Pools.* An item pool is one of the key components of the system. In the item pools, each level of knowledge includes a lot of items which have verification, and each item includes difficulty, discrimination, exposure frequency, belongs the domain, keywords, and other information.

*(3) Items Generation Module.* The module is based on the level of temporary knowledge of learner and item parameters, such as difficulty, discrimination, guessing coefficient, and according to multiple criteria and usage of the simulated annealing algorithm for selecting the right items.

*(4) Temporary Learner Model.* The system creates a temporary learner model for learners and dynamically updates the testing process. On the one hand, it establishes the likelihood function of learners on item response, uses Newton-Raphson iteration method for solving the likelihood function, and calculates maximum likelihood estimates; on the other hand, the model is used to generate items.

*(5) Test Termination Criteria.* The test termination criteria use three criteria combined which are the standard deviation of estimated level of knowledge to reach threshold *e*, test time, and maximum test length.

### 4.2. Test Procedure

**Figure 3:**
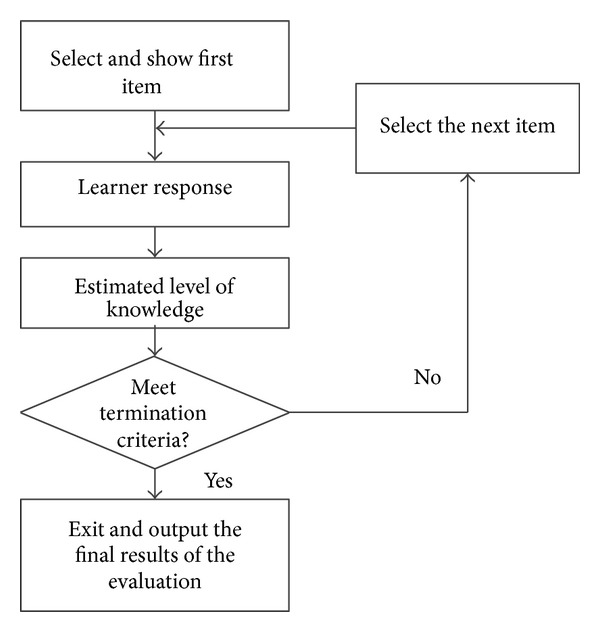
Adaptive testing flow chart.

The test process is shown in Figure 3, which includes the following basic steps.

**Step 1 (the generation of first item)**

Based on the basic information of learners, the system randomly selected first item from the item pool.

**Step 2 (the estimates of learners' knowledge level)**

The knowledge level of the learner's reestimates uses the* Newton-Raphson* iteration method.

**Step 3 (intelligent item selection)**

According to the learners' answer for items as well as the update ability values, the item generated model based on multiple criteria and adaptive select items which are appropriate to their level of knowledge of the learner.

**Step 4 (stop criterion)**

If test termination criteria are not met, then return to Step 2 and select the appropriate test item for the learners; otherwise, end the test and show the results.

### 4.3. System Implementation

**Figure 4:**
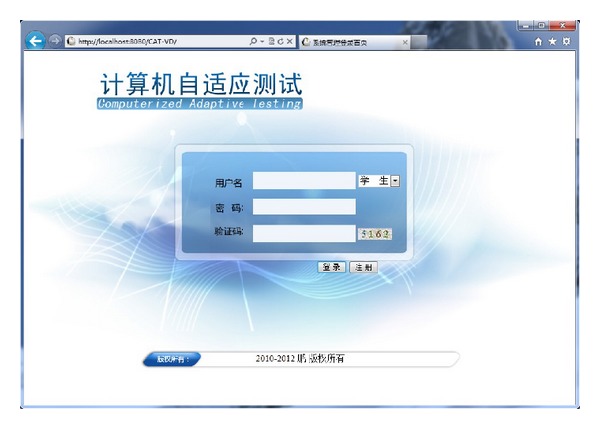
Log home.

**Figure 5:**
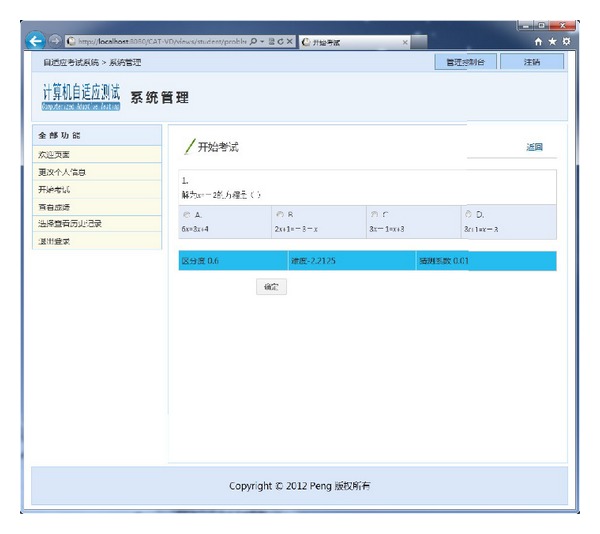
Web-based testing environment student's side.

On implementation, the prototype system used tools containing Myeclipse 6.0, Rational Rose 2003, MySQL 5.0, Powerdesigner 15, Tomcat, and so forth. The login home and student's side are shown in Figures 4 and 5.

System implementation was done by developing a system prototype to experimentally research and analyse the performance of our proposed items generation system with memory-based simulation algorithms. And according to the result the system is improved.

## 5. Experimental Analysis

In order to evaluate the performance of web-based testing environment, this study conducted a series of experiments. The experimental environment is Inter Core 2 2.0 GHz, 2 G RAM, 250 G hard disk, and 5400-RPMaccess speed. To analyze and compare the performance of several experiments, four item pools were constructed; the numbers of items were 100, 250, 500, and 691.  Table 1 shows the characteristics of each item pools.

In the following experiments, test including 10 topics and weights were *w*_1_ = 0.05, *w*_2_ = 0.1, *w*_3_ = 0.1, *w*_4_ = 0.15, *w*_5_ = 0.15, *w*_6_ = 0.15, *w*_7_ = 0.1, *w*_8_ = 0.1, *w*_9_ = 0.05, and *w*_10_ = 0.05. The numbers of iterations of function of the temperature decreases were 5, 10, 15, and 20, respectively.

On the efficiency of items selection, the execution time to SA search, exhaustive search, and random search method were compared to implement the SA algorithm assessment. The experiment is performed 10 times on each of item pool of three methods.  Table 2 shows the results of average execution time of selecting an item from each of the item pools.

**Figure 6:**
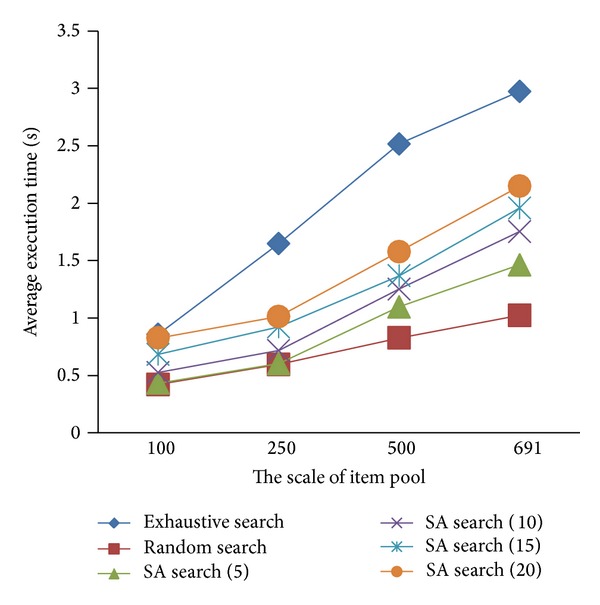
Algorithm execution time comparison.

Figure 6 shows the average execution time of SA search, exhaustive search, and random search, and SA search uses different iterations. From the figure we can see that item selection of the time of each method is constantly increasing with the increase of the scale of the item pool, but the degrees of growth are different. When the number of items is less than 100, the execution time of the SA search algorithm which we proposed is relatively close to the exhaustive search. When the number of items is larger than 250, the execution time of the SA search algorithm is less than the exhaustive search. The increase of exhaustive search is relatively large, mainly due to its need to scan item pool every time of item selection. The average selection time of SA search method is lower than the exhaustive search proposed in this study mainly because of the method using simulated annealing algorithm which reduces the number of comparisons between the items as well as reduces the number of the calculation of the items.

The time complexity of SA search and exhaustive search to obtain the optimal solution is analyzed as follows. The time complexity of an exhaustive search is *T*(*n*) = *O* (*N*), where *N* is the number of item and indicates that the execution time of enumeration search method is increased with the expansion of the scale of item pool. The time complexity of an SA search is *T*(*n*) = *O*(*kL*_*m*_ *t*(*n*)), where *k* is the number of iterations, *L*_*m*_ is maximum length of *k* Markov chain, and *t*(*n*) is a polynomial function of the size *n* of the item selection problem. Therefore, *T*(*n*) = *O*(*kL*_*m*_ *t*(*n*)) indicates that the execution time of SA search is almost independent of the number of items and can search approximate optimal solution in polynomial time. These results show that when dealing with large-scale item pools, the SA searches more efficiently than an exhaustive search.

**Figure 7:**
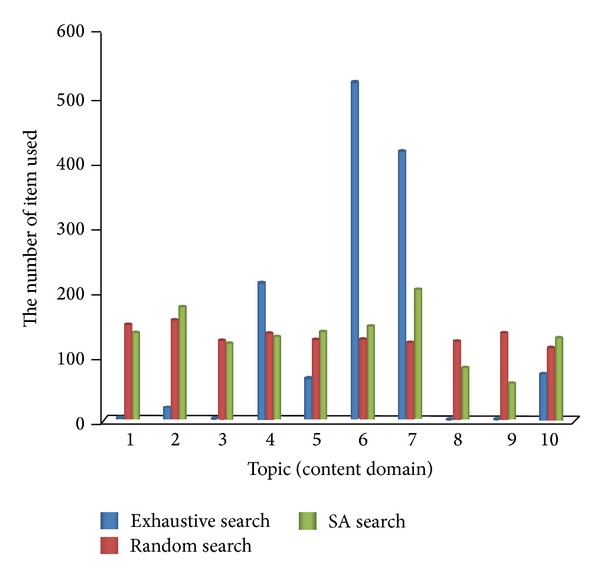
Content balance comparison.

Figure 7 is the experimental results in the content balance of exhaustive search, random search, and SA search (cooling 20). As can be seen from the figure, the exhaustive search method chooses the items focused on topic four, topic six, and topic seven and the maximum number of 520; however, the number of items chosen on other topics is relatively small and even never selected in topic three and topic eight, so there is a serious problem for content balance. The random search method selected items from item pool that are random at every time and were selected items in each topic. Therefore, it did not have the problem of unbalanced content. And the SA search method selected items in various topics, which in topic two and topic seven it selected more items, up to 201, and selected items in topic eight and topic nine were fewer; this is mainly due to the number of items for each topic being uneven. Comparison shows that relative to the exhaustive search method, the SA search method is more balanced on the test content. Therefore, a comprehensive evaluation of the learners is more reasonable.

In addition, the number of exposure times of each item was statistical and the results are shown in Table 3. As can be seen, with respect to the exhaustive search method, usage of the SA search method (cooling 20) for item exposure was better controlled and improved test safety.

**Figure 8:**
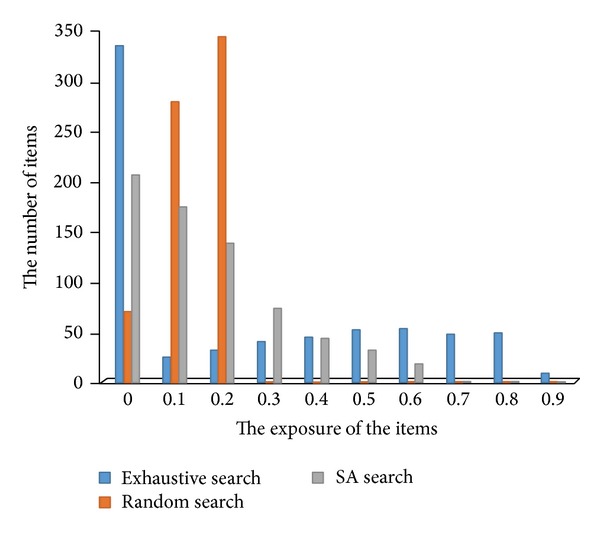
Number of items selection comparison.

Figure 8 is a comparison of three methods in the result of item exposure. As can be seen from the figure, the exposure based on the exhaustive search method is relatively high, and parts of items even reached 0.9. And all items are uniform in exposure when using random search. And when using the SA search method, the exposure of most items is less than 0.3, but there is a small part of the high exposure of the items. Through analysis we can see that SA search method greatly reduces the exposure of items and increases test security.

As can be seen from the results of the above experiments, the SA method is very efficient and easily performed. While maintaining high accuracy, it can be a very good exposure control for items, and it improves test security and ensures balanced content.

## 6. Conclusion

Switching from the traditional learning environment to the adaptive, intelligent, and personalized e-learning environment is rapidly changing in the world; the fundamental reason is to provide learners with an individualized learning environment. In addition, the application of adaptive assessment in the e-learning environment also increases personalization.

In this paper, we demonstrated more innovative and more personalized environment and proposed a web-based testing environment which uses a simulated annealing algorithm with memory and meets a variety of evaluation criteria, in order to deal with a computerized adaptive testing that faced part of the items high exposure, test content imbalance, lower efficiency of the system, and other issues in the application. We analyzed a series of experiments on the system performance; experimental results show that in ensuring a reasonable case execution time, the system can choose a near-optimal test items from the items pool for each learner and in the items exposure control and content balancing also can make fairly good results.

The adaptive evaluation module integrated into the web-based testing environment can assess learners based on their level of ability. Therefore, the result of this study is to contribute to the development of the test environment and e-learning, and making adaptive assessment will be more efficient. In the future research, we will use the data mining technology to discover the hidden relationship between items or between items and the learners. The results obtained are applied to the selection of items and provide learners with more scientific and reasonable items; in addition, we will use fuzzy inference methods in the learner model, reasoning their knowledge level.

## Figures and Tables

**Algorithm 1:**
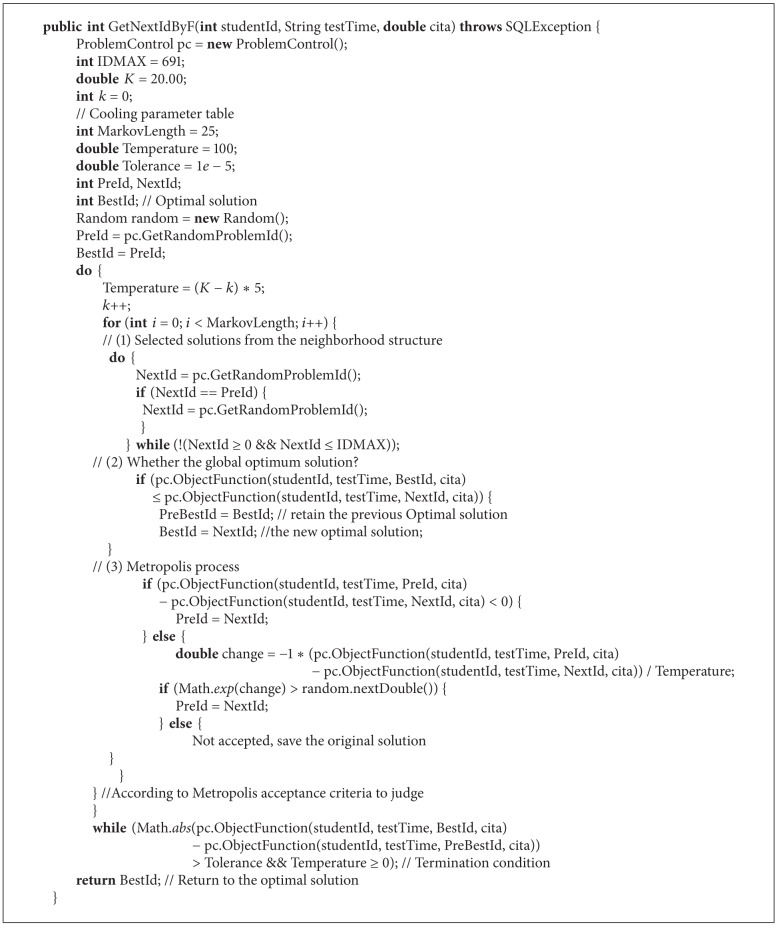
Java code description of the algorithm.

**Table 1:** Item pools scale and parameters description.

Item pool	Item numbers	Average discrimination	Average difficult	Average guess factor
1	100	0.78	0.56	0.25
2	250	0.69	0.53	0.25
3	500	0.71	0.59	0.25
4	691	0.74	0.55	0.25

**Table 2:** The average execution time of the algorithm.

Item numbers	Average execution time (s)					
	Exhaustive search	Random search	SA search			
			5	10	15	20
100	0.857	0.421	0.433	0.524	0.682	0.826
250	1.648	0.596	0.602	0.717	0.921	1.012
500	2.515	0.826	1.096	1.251	1.367	1.578
691	2.973	1.022	1.463	1.752	1.958	2.147

**Table 3:** Comparison of exposure.

	Exhaustive search	Random search	SA search
Mean	0.13	0.02	0.08
Maximum	0.90	0.20	0.60
Minimum	0.00	0.00	0.00
Overexposure percentage (%)	48.00	6.00	24.00
Never exposed percentage (%)	48.30	10.10	30.20
Maximum number of exposure of each test	19.00	2.00	3.00
